# Presentation of Acrodermatitis Chronica Atrophicans Rashes on Lyme Disease Patients in Canada

**DOI:** 10.3390/healthcare8020157

**Published:** 2020-06-04

**Authors:** John D. Scott

**Affiliations:** International Lyme and Associated Diseases Society, 2 Wisconsin Circle, Suite 700, Chevy Chase, MD 20815-7007, USA; jkscott@bserv.com; Tel.: +1-519-843-3646

**Keywords:** Lyme disease, *Borrelia burgdorferi* sensu lato, rashes, acrodermatitis chronica atrophicans, symptoms, persistence, ticks, Canada

## Abstract

Lyme disease (Lyme borreliosis) is a complex multisystem illness with varying clinical manifestations. This tick-borne zoonosis is caused by the spirochetal bacterium, *Borrelia burgdorferi* sensu lato (Bbsl) and, worldwide, presents with at least 20 different types of rashes. Certain cutaneous rashes are inherently interconnected to various stages of Lyme disease. In this study, five Canadian Lyme disease patients from a multi-age range presented various phases of the acrodermatitis chronica atrophicans (ACA) rash. In each case of ACA, the underlying etiological pathogen was the Lyme disease spirochete. Although ACA rashes are normally found on the lower extremities, this study illustrates that ACA rashes are not directly correlated with a tick bite, geographic area, age, Bbsl genospecies, exercise, or any given surface area of the body. Case 4 provides confirmation for an ACA rash and gestational Lyme disease. One patient (Case 5) puts forth a Bbsl and *Bartonella* sp. co-infection with a complex ACA rash. This study documents ACA rashes on Lyme disease patients for the first time in Canada.

## 1. Introduction

Lyme disease (Lyme borreliosis) is a multisystem, zoonotic infection caused by the bacterium, *Borrelia burgdorferi* sensu lato (Bbsl) [1]. Worldwide, there are at least 24 genospecies in the Bbsl complex. Several of these Bbsl genospecies are known to be pathogenic to humans. In continental North America, at least eight Bbsl genospecies have pathogenic potential, including *B. afzelii* [2,3], *B. bissettiae* [4,5,6,7,8,9,10], *B. burgdorferi* sensu stricto [1,3,11], *B. californiensis* [3,6], *B. garinii* [3,11,12], *B. kurtenbachii* [13,14,15], *B. mayonii* [16], and *B. spielmanii* [3,17,18]. European countries have three other Bbsl genospecies that are determined to be pathogenic to humans, namely *B. bavariensis* [19,20], *B. lusitaniae* [21,22], and *B. valaisiana* [23,24]. Globally, Lyme disease has been recognized in 85 countries [25], and evidence abounds on the wide dispersal of Lyme disease vector ticks by songbirds [25]. Most significantly, this tick-borne zoonosis can have a broad spectrum of clinical manifestations that often involve varied, cutaneous rashes.

Acrodermatitis chronica atrophicans (ACA) is one of the rashes associated with Lyme disease. ACA is a chronic skin lesion that is normally seen on the extremities, and starts with bluish-red discoloration and, years or decades later, may develop into the latent, atrophic phase [26]. From the time of tick bite or occurrence of initial infection, the development of ACA lesions may be several years [26]. ACA lesions often develop slowly, and the most common site for an ACA rash is the leg [27]. Joint or bone involvement may occur underneath the ACA skin lesion. Some patients with ACA rashes have episodic attacks or joint effusions of the knee [27]. They may manifest in painful occurrences in different parts of the leg, including the knee, foot and ankle. ACA does not heal spontaneously, but may lead to atrophy, sclerosis and ulceration. The majority of patients with ACA experience peripheral neuropathy [28], whereas others may have severe localized pain. Swelling or pain often occur in the affected area. Some ACA patients complain of musculoskeletal pain while a few experience knee arthritis and/or synovitis. 

The ACA rash was first recognized in Europe, but the causal microorganism was not discovered until a century later. The ACA rash was first described by Buchwald in 1883 in Germany [29], and the first case reports of ACA in North America date back to 1895 [30,31]. Lavoie et al. documented an ACA as a late manifestation of Lyme disease in the U.S.A. [32]. Additionally, Kaufman et al. reported the first cases presenting with an ACA in North America that had an interconnecting link to a Lyme disease endemic area (eastern Long Island, New York) [33]. Canadians do not have to frequent a Lyme disease endemic area [34]; they can contract this zoonosis at any one of a hundred known hotspots across Canada. Biogeographically, ACA has been described in the northern, central, and eastern parts of Europe, especially countries bordering the Baltic Sea and, to a lesser degree, in North America [27]. Even though ACA symptomatology has been present in Canadian inhabitants for many years, and predate the discovery of the Lyme disease bacterium, we now provide the first case reports of ACA rashes in Canada. 

The etiology of the ACA rash was not confirmed until 1983 [35,36,37]. In the Baltic states, *B. afzelii* is the predominant Bbsl genospecies, and the castor bean tick, *Ixodes ricinus* (Acari: Ixodidae) is the zoonotic vector [35,38]. East of the Rocky Mountains, the blacklegged tick, *Ixodes scapularis*, is known to harbour and transmit any combination of at least 9 different tick-borne pathogens, including Bbsl and *Bartonella* spp. [38]. Pathologically, *B. afzelii* has been cultivated from ACA lesions, and some of these patients had negative serology [36]. ACA may be the first sign of borrelial infection; however, many patients have other preliminary and secondary stage manifestations [39]. Approximately one-third of patients with an ACA recall a tick bite. An erythema migrans (EM) rash can last 2 years while an ACA rash can last 10 years or more [36]. Epidemiologically, *B. afzelii* has recently been detected in North American patients [3].

Advanced ACA rashes may have fibrous thickening of the skin. Of those with cutaneous involvement, there has been an association between ACA and peripheral neuropathy in the limbs [40]; some may be recrudescent or be ongoing. Some patients may also experience intermittent lightning pains in extremities. Central nervous system disturbances may occur in patients with ACA. Profound fatigue and personality changes may accompany an ACA.

In the early phase of ACA, there is an inflammatory reaction with varying degrees of edema (excessive fluid in subcutaneous tissues). As a result, the epidermis becomes thin followed by a degeneration of collagen and elastin tissue [27]. After full-term antibiotic treatment, the ACA rashes normally disappeared gradually. Co-infections of tick-borne diseases, which are common, may result in unusual skin patterns. 

## 2. Methods

### 2.1. Participant Selection

Each participants was required to serologically test positive for Lyme disease using a Clinical Laboratory Improvement Amendments (CLIA)-certified laboratory, and have clinical symptoms of Lyme disease, and be a confirmed case. 

### 2.2. Informed Consent

All subjects or their parents gave informed consent to participate in this dermatological Lyme disease study.

### 2.3. Photos of ACA Rashes

Photos of ACA rashes plus background information is provided for each participant. In order to maintain patient confidentiality, photo credits have been withheld.

### 2.4. Cultures and Molecular Testing

The skin lesion on Case 4 was sampled using a 3-mm skin biopsy punch, and skin biopsies were inserted directly into vials of Barbour–Stoenner–Kelly (BSK) II medium, and sent by courier to the Division of Medical Microbiology, Department of Pathology, University of British Columbia, Vancouver, British Columbia. Upon their arrival, the culture tubes were incubated at 34 °C, and checked weekly for spirochetal activity. In order to avoid culture contamination, polymerase chain reaction (PCR) amplification was conducted in a separate filtered laboratory. PCR testing identifies the genes of Bbsl, namely OspA, 16S rRNA, and HSP60. The OspA (31 kilodalton (kDa)), OspB (34 kDa), OspC (22–25 kDa), P39 (39 kDa), flagellin (41 kDa), P22 (22 kDa), and P93 (93 kDa) bands were profiled using sodium dodecyl sulfate-polyacrylamide gel electrophoresis. These isolates were further analyzed for plasmid profiles; pulsed-field gel electrophoresis (PFGE) separation of macro-restriction digests (*Mlu*I) and (*Sma*I) of total DNA were applied.

### 2.5. Subject Profiles

The clinical profiles of the five study subjects with ACA rashes are provided below. The Western blot IgM and IgG results for all subjects are given, and PCR (polymerase chain reaction) outcomes are made available for select subjects.

#### 2.5.1. Case 1

**Figure 1:**
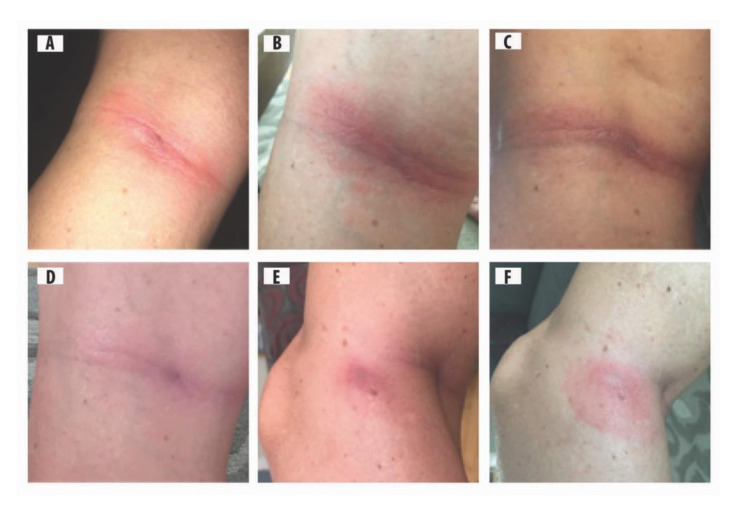
Middle-aged female with two types of rashes on left knee: (**A**) homogeneous rash with a punctum in the centre, 12 d after tick bite; (**B**) early inflammatory phase of acrodermatitis chronica atrophicans (ACA) rash, reddish-blue discoloration with punctum, 14 d after tick bite; (**C**) early inflammatory phase of ACA, more pronounced, extensive purple discoloration, 24 d after tick bite; (**D**) purple discoloration fading as spirochetes disseminate, 52 d after tick bite; (**E**) second rash, homogenous type, on inside of left knee with partial reddish-blue discoloration, punctum from tick bite is apparent; and (**F**) second rash, homogeneous type, purple discoloration fading, and punctum evident.

A middle-aged, Canadian female went on a bicycle tour through Scandinavia (i.e., Lithuania, Latvia, Estonia, and Finland), and developed two rashes. Although not recalled, it is believed that she was bitten by ixodid ticks in late June in Estonia. A rash appeared within 2 wk of tick bite on the back of the left knee, and the punctum (point of tick hypostome entry) in the centre of the skin rash suggests that she had been bitten by a tick that became fully engorged, and dropped off (Figure 1). Morphologically, a tick is the only arthropod that has a piercing, anterior-pointing mouthpart with backward-pointing barbs (denticles), and leaves a visible punctum. The second rash, on the inside of the same knee, was typical of an erythema migrans (EM) rash, and the punctum in the centre of this rash suggests that she was bitten by another tick that had acquired a blood meal (Figure 1). 

Within 2.5 mo after tick bite, the Lyme IgM/IgG enzyme immunoassay (EIA) was reactive. As well, the IgM Western blot was reactive. Three months later, further serological testing for Lyme disease was conducted and, similarly, the EUROIMMUN *Borrelia* IgG immunoblot was positive for *B. afzelii*. At this time, clinical manifestations included fatigue, joint and muscle pain, brain fog, headaches, and poor sleep. At no time did this patient experience any night sweats, chills, or profound fatigue, which would be suggestive of human babesiosis or piroplasm infection. 

Three mo after the two tick bites, she was administered a course of doxcycline, 100 mg bid, for 21 d, and the rash disappeared. As follow-up, 4.5 mo after tick bite, a second round of doxycycline, 100 mg bid, was started for 28 d. Although symptoms (i.e., fatigue, brain fog, headaches, and poor sleep) improved somewhat, her symptoms did not resolve. Subsequently, 6 mo after tick bite, she was administered intravenous (IV) ceftriaxone, 2 g qd, for 30 d; the pain in the knee joint persisted. Seven months after tick bite, a second course of IV ceftriaxone, 2 g qd, commenced for 28 d. At the conclusion of her second IV ceftriaxone treatment, she still experienced pain in her left knee. This patient will continue to be monitored, and follow-up antibiotic treatment may be administered. 

#### 2.5.2. Case 2

**Figure 2:**
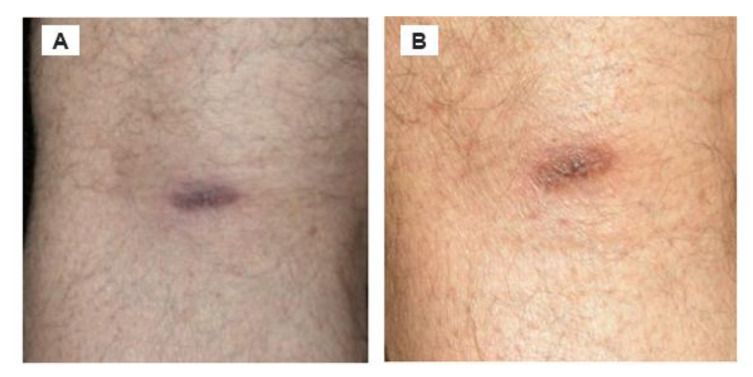
ACA located on the posterior side of left knee, and there was no recent involvement of a tick bite. (**A**) Rash when noticed. (**B**) Rash 4 months later. Of note, the synovial membrane and synovial fluid of the knee are prime foci for sequestration of Lyme disease spirochetes.

A 72-year-old Canadian male had re-occurring flu-like symptoms, and consulted 12 different healthcare practitioners (i.e., infectious disease specialists, internal medicine specialists, neurologists, psychiatrists, and rheumatologists) over a period of 4 years before he received a confirmed diagnosis of Lyme disease. The patient was not able to break out of this cyclically febrile pattern and, retrospectively, he had late stage Lyme disease, signifying chronic Lyme disease. Over the following 30 years, he received multiple regimens of antimicrobials, including tetracycline, doxycycline, ceftriaxone, amoxicillin plus probenicid, clarithromycin, intramuscular benzathine penicillin G, and disulfiram. Despite multiple regimens of antimicrobial therapy, this patient continues to remain symptomatic with signs and symptoms of chronic Lyme disease. After 30 years, the patient developed an ACA rash on the back of his left knee (Figure 2). This was recognized as a clinical diagnosis supported by Lyme disease serology consisting of ELISA and Lyme Western blot (i.e., IgM, IgG) and Lyme Immunoblot IgM and IgG. In addition, spirochetes were cultured from fresh semen and, using PCR and DNA sequencing, this isolate was positive for *B. burgdorferi* sensu stricto ([41], Case 10). This Bbsl genospecies is pathogenic to humans.

At one point, this patient developed encephalopathic symptoms, including brain fog, short-term memory loss, speech difficulties, inability to concentrate, short attention span, and dementia (Figure 2). As a result, he lost his physical stamina, his professional career, and his ability to drive a vehicle. With ongoing antibiotic treatment, he was able to obtain restoration from cognitive impairment, and was able to drive again. The improvement period for Case 4 was slow, and he lived a restricted, reclusive lifestyle.

#### 2.5.3. Case 3

**Figure 3:**
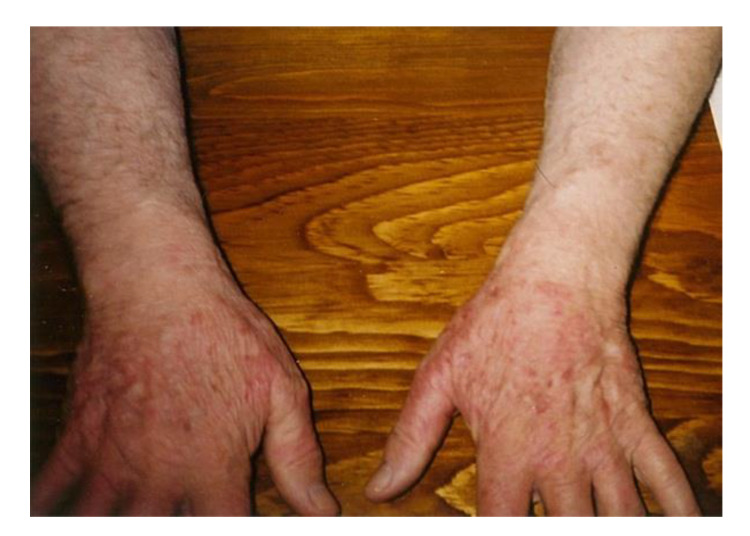
Rash on hands with reddish-blue discoloration is consistent with ACA. The discoloration resolved temporarily with antibiotic treatment, and then recrudesced.

A 71-yr-old Canadian male, who had immigrated from Hungary, developed an ACA rash with bluish-red discoloration on both hands (Figure 3). This patient had arthritic symptoms, and was repeatedly treated with doxycycline, 100 mg bid. Overall, he was treated for 2 mo each round, and continued for 9 rounds, 18 mo in total, and his symptoms improved each time. However, when his arthritic symptoms flared up again, he had Lyme disease serological testing, and his lab results were consistently positive using the two-tier Lyme disease serology testing. Consistent with the majority of Lyme disease patients, this patient exhibits persistent infection due to Lyme disease spirochetes.

With antibiotic treatment this patient was able to function in society with reduced stamina. Without fail, when he was taken off antibiotic treatment, his arthritic symptoms exacerbated. The presence of re-occurring discoloration of the hands, suggests that Lyme disease spirochetes sequestered in the collagenous tissue of his hands despite regimens of standard doxycycline treatment (Figure 3). This recrudescent borrelial infection elucidates a clear-cut case of chronic Lyme disease. This patient consistently tested positive for Lyme disease after each round of standard antibiotic treatment. Re-occurrence of symptoms underscores the persistence of this stealth pathogen in the human body. He experienced a pattern of recrudescence between each remission period and each antibiotic treatment. In all, he was able to retain quality of life, and enjoy his wife and grandchildren. 

#### 2.5.4. Case 4

**Figure 4:**
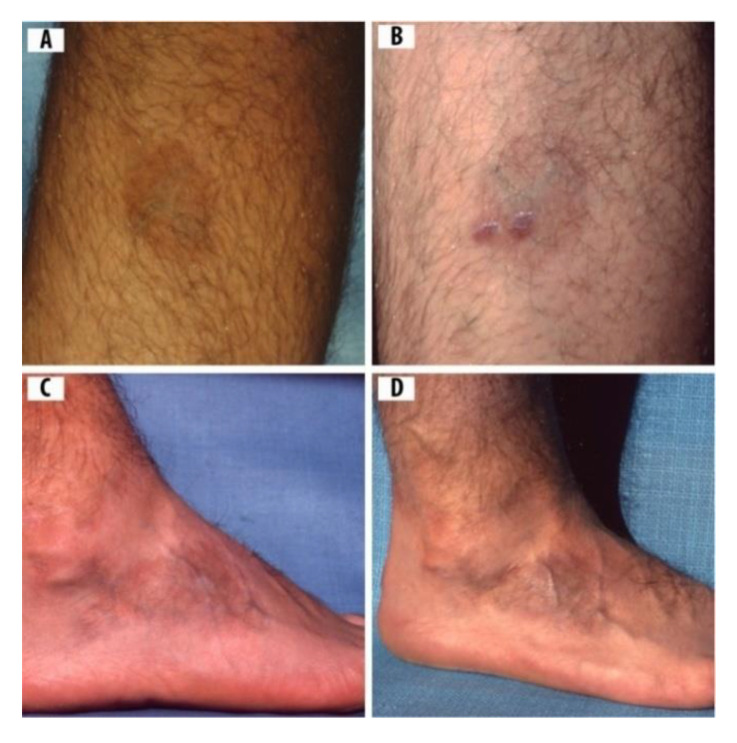
A 14-yr-old boy with rashes compatible in appearance with ACA: (**A**) ACA rash on the calf of right leg before skin biopsies; (**B**) skin biopsies test positive for *Borrelia burgdorferi* sensu lato (Bbsl) by polymerase chain reaction (PCR); (**C**) ACA on right foot with pronounced swelling and reddish-blue discoloration; and (**D**) hyperpigmented, atrophic ACA on right foot.

A 14-yr-old Ontario boy who had no history of out-of-province travel, developed reddish-blue lesions on his right calf (viz. gastrocnemius muscle) and on his right foot (Figure 4). Using a 3-mm skin biopsy punch, the attending physician excised 2 tissue samples from the lesions on the calf area, and inserted them in Barbour–Stoenner–Kelly (BSK) II culture medium, as described above. These two skin biopsies were positive for Bbsl by immunochemistry and, using PCR, were positive for *B. burgdorferi* sensu stricto. As well, this boy was positive for Lyme disease using the two-tier Lyme disease serology testing. The Lyme Western blot (i.e., IgM, IgG) tests were positive for *B. burgdorferi* sensu stricto. Initially, as an infant, his family physician deemed his feet to be normal. 

As a toddler, however, a shoe store clerk noted an abnormality in his feet. Subsequently, upon referral, an orthopedic surgeon considered the misshapen feet to be a idiopathic deformity. One foot was corrected with multiple casting, whereas the other foot had to be straightened with orthopedic surgery. Corrective shoes, which toed out, were used to straighten the metatarsal curvature. Later, as the result of a job-related transfer, a family physician in another town hastily misdiagnosed his lesions as "burn scars" (Figure 4). After the last casting, another orthopedic specialist was consulted, and he recommended wearing orthopedic shoes for five years, and the metatarsal curvature was corrected. After careful searching, a clinician, who was knowledgeable about Lyme disease, correctly diagnosed the ACA rashes, and treated this patient with doxycycline, 100 mg bid, consisting of 2 mo sequelae totaling three years. This young boy experienced severe swelling and osteomyelitis in his right foot (Figure 4). With long-term antibiotic treatment, his feet returned to normal. The symptoms of Case 4 cleared up, and he was fully recovered after 3 years of antibiotic treatment.

Both the mother ([41], Case 9) and father ([41], Case 10) of Case 4 have definitive proof of chronic Lyme disease, as previously described [41]. The parents were both PCR-positive for *B. burgdorferi* sensu stricto. Not only was the filial son born with bilateral clubfeet, his lower leg skin biopsies were PCR-positive for *B. burgdorferi* sensu stricto, as described above. However, since the clubfeet predated the discovery of the etiologic microorganism, Bbsl, the attending clinicians likely failed to diagnose this boy as a pediatric case of Lyme disease. This pediatric case dates back to birth in 1976, and probably constitutes the first documented case of transplacental Lyme disease in Canada. It is noteworthy that the mother and father and their biological son were all positive for *B. burgdorferi* sensu stricto.

#### 2.5.5. Case 5

At presentation, a 64-yr-old Canadian male had a sore on his right leg with a erythematous-ACA rash. The rash was sore and would not heal. His healthcare practitioner administered antibiotic treatment that included cloxacillin, 250 mg bid, for 7 d. Since the rash had not resolved, he was again treated with cloxacillin, 250 mg for 14 d. His quality of life improved, and he returned to his professional career that entailed frequent outdoor work. 

Three years later, he had another clinical assessment, and was tested for Lyme disease and human bartonellosis. The Lyme disease Western blot IgM and IgG were positive and, likewise, the *Bartonella* IgM and IgG were positive. He was prescribed a concurrent antibiotic regimen: clarithromycin, 500 mg bid; cefdinir, 300 mg bid; minocycline, 200 mg bid; hydroxychloroquine, 200 mg bid; and pantoprazole, 40 mg qd for 30 d. He followed up his antibiotic therapy with a herbal program of Japanese knotweed, cordyceps, capucha mushroom, and other herbal combinations that included quercetin, resveratrol, and chaga tea. 

**Figure 5:**
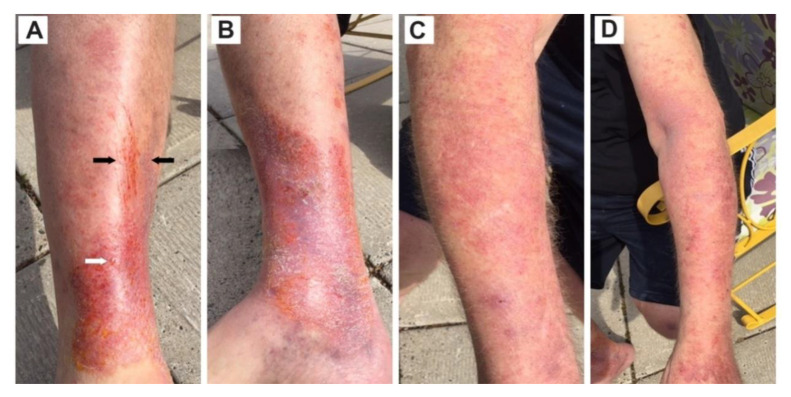
A 64-yr-old male exhibited rashes encompassing a set of co-infections, namely ACA, blister exudate, erythematous rash, and *Bartonella* streaks (striae). (**A**) Right leg, front view, compound rash with *Bartonella* striae concomitantly embedded in an inflammatory atrophic ACA at the ankle. The black arrows point to *Bartonella* striae, and the white arrow indicates exudate droplet typically produced by a blister rash. (**B**) Right leg, inside view, inflammatory atrophic ACA at foot. The orange discoloration is the result of an application of chlorhexidine antiseptic. (**C**) Right arm, diffuse erythematous rash graduating to an inflammatory ACA rash at wrist. (**D**) Left arm, diffuse erythematous rash progressing to inflammatory atrophic ACA at hand.

Six years later, he presented to his healthcare practitioner with complex rashes on four extremities. This patient experienced considerable itching with his rashes. The main symptoms included undulating fatigue, numbness in hands and feet, tingling in fingers, swelling in appendages and digits, moderate arthritis, and muscle ache. Additionally, this patient had numbness and swelling in extremities (Figure 5), and related itching all over his body. The exudate droplet on this right leg (Figure 5), provides suggestive evidence of ulceration.

Overall, it was 9 years from the time of the initial rash, that Case 5 received disulfiram, 125 mg, once every third day, for 30 d, and monitored efficaciously for organ (liver, kidney) function. A probiotic was taken separately from his medication to rejuvenate gut microbiome. The patient gradually improved, but he experienced ongoing fatigue as the body detoxified the pathogen breakdown by-products, namely borrelial and *Bartonella* biotoxins. Of particular note, the target dosage of disulfiram varies greatly depending on body weight, chronological age, medication tolerance, and physiology of the individual patient.

## 3. Results

Within 2 weeks, the culture medium for Case 4 revealed characteristic spirochetes by dark-field microscopy. Sodium dodecyl sulfate-polyacrylamide gel electrophoresis analysis of these isolate was compared to the B31 type strain of *B. burgdorferi* sensu stricto. Molecular testing, using PCR, detected genes of Bbsl, namely OspA, 16S rRNA, and HSP60. In addition, a lucid 135 kbp PFGE band, which is characteristic of *B. burgdorferi* sensu stricto, was detected in the restriction digest. The isolates also reacted with monoclonal antibodies of OspA, OspB, OspC, BmpA (P39), flagellin, P22, and P93. With the exception of flagellin, these protein bands are species-specific for Bbsl; flagellin is a *Borrelia*-specific band for the Lyme disease spirochete. Based on the above tests of the skin biopsy isolates (Case 4), the spirochetes were confirmed as *B. burgdorferi* sensu stricto, which is pathogenic to humans.

## 4. Discussion

### 4.1. Pathogenesis of ACA Rash

**Figure 6:**
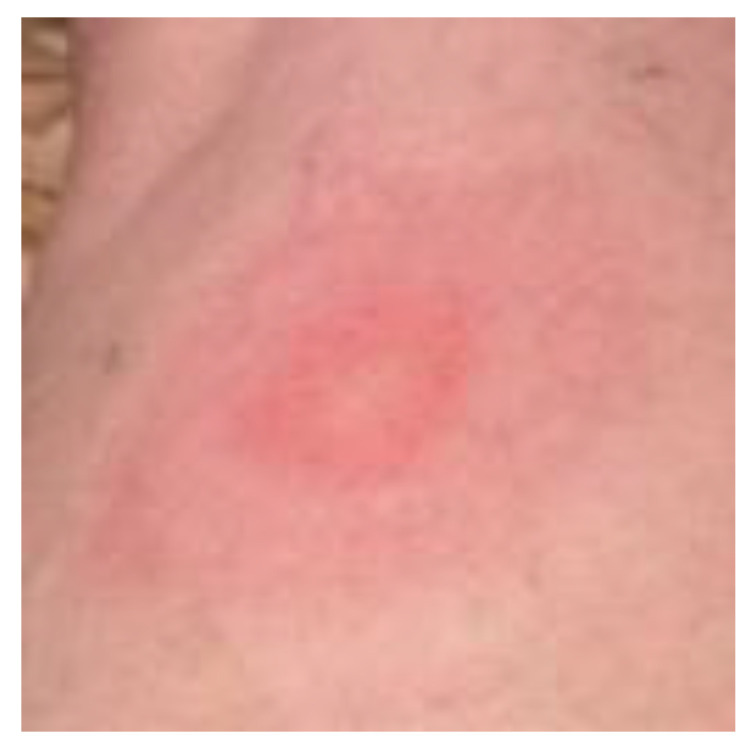
Erythema migrans, a ring-shaped or bull’s-eye rash on the upper leg of a middle-aged woman. This erythematous lesion is a classic erythema migrans (EM) rash.

Rashes can be prominent clinical manifestations of Lyme disease, and appear at almost any age. Although the classic EM rash, called a bull’s-eye rash (Figure 6), is fixed in the minds of many people, there are at least 20 different types of rashes (A.B. MacDonald, Pathologist, personal communication) and, of these rashes [27,39,42,43,44,45,46,47], ACA sporadically presents in Lyme disease patients. The classic EM rash is more easily recognized, but may be misdiagnosed if it is vesicular, necrotic or otherwise atypical in appearance. Based on several studies, only 9–39% of Lyme disease patients have a rash and, of those with an EM rash, more than 50% have a homogenous rash [47,48,49,50]. In individual cases, the EM rash may spread over the entire body in four months, and appear as diffuse reddening and/or edematous swelling [39]. The tick bite may go unnoticed; however, a cutaneous rash may form. Lyme disease spirochetes normally disseminate radially through the epidermis and, also, via the vascular and lymphatic systems. In one particular study of EM and ACA rashes in central Europe, only 2 (7%) of 28 patients recalled a tick bite [51]. 

Lyme disease rashes can be atypical, or appear as multi-centric erythema migrans denoting hematogenous spread in the body. In all five cases of the present study, patients continued to encounter active Bbsl infection after standard antibiotic treatment. Of significance, two patients had formidable molecular evidence of *B. burgdorferi* sensu stricto, which is pathogenic for Lyme disease. The clinical and morphological features and molecular testing associated with EM and ACA rashes in the present study are consistent with proven EM and ACA rashes published in the scientific literature.

### 4.2. Early and Late ACA Rashes

Cutaneous rashes have an interconnecting link to clinical manifestations. ACA has three phases: an early inflammatory stage, a late inflammatory stage with atrophy, and a final latent atrophic stage without inflammation [52]. Often the early stage is overlooked in the scientific literature. The early inflammatory phase is exhibited by Case 1 (Figure 1b,c). Although there is no skin biopsy from the back of the knee (Case 1), there is substantive evidence of effusion of blood incorporated in the cutaneous tissue surrounding the tick bite. As previously stated, Case 1 tested positive for *B. afzelii*. Pathologically, *B. afzelii* is one of the etiological agents of ACA rashes [2]. Pertinent to Case 1, the initial stage of an ACA is visible, and shows the dissemination of spirochetes radiating from the punctum. An ACA rash gradually intensified its purplish color, and then decreased in intensity. These eruptions are consistent with the early stages of an evolving ACA lesion. Moreover, the right knee of Case 1 has no discoloration, and it had exactly the same amount of exercise as the left knee; the photographic pattern and presence or lack of pain are not bilateral. It is most likely that the effusion and purplish lesion in Case 1 resulted from spirochetosis, and not exercise. 

Chang et al. detected Bbsl in the synovial membranes of dogs, which indicates persistent infection. It also suggests that the enduring inflammatory pain experienced by Case 1 represents concomitant evidence of clinical arthritis or synovitis (inflammation of a synovial membrane) caused by Lyme disease spirochetes [53]. These findings suggest that Bbsl side-steps the immune system, and survives proficiently within connective tissue. As a result, the synovial membrane can act as a sanctuary for Bbsl spirochetes, protecting these microbes from antibiotics and/or immune response. The ACA rashes on the leg of Case 4, which were recognized after his infant-childhood growth spurt, affirm gestational Lyme disease acclaimed in other neonates [54].

### 4.3. Combinations and Co-Infections

In the present study, Case 5 has a combination of Lyme disease and human bartonellosis. The patient exhibited a combination of rashes on the upper and lower extremities of the body (Figure 5). *Bartonella* streaks were concomitantly embedded in an inflammatory atrophic ACA rash (Figure 5). *Bartonella* often displays a parallel, streaky pattern that unfolds a distinguishable characteristic of human bartonellosis. *Bartonella* sp. infection is commonly associated with Lyme disease. The rash aggregates (i.e., ACA, blister-like exudate droplets, *Bartonella* sp. streaks, and diffuse erythema rashes) on the left leg of Case 5 elucidates a co-infection of Bbsl and *Bartonella* sp. Although Lyme disease and human bartonellosis are frequent co-infections, they are recalcitrant tick-borne pathogens to treat. Standard combinations of antimicrobials often suppress the symptoms but commonly do not permanently clear these infections. *Bartonella* spp. are among the most efficient stealth pathogens because these bacteria can invade erythrocytes, macrophage, dentritic cells and endothelial cells [55]. The combination of ACA, streaking, and blister exudate is fundamentally a combination of three different types of rashes culminated in co-infection of Bbsl and *Bartonella* sp. Based on the findings of Goldberg et al. [56], the exudate droplets on the right lower leg of Case 5 could be harboring Bbsl spirochetes and/or *Bartonella* sp. bacteria. 

### 4.4. Differential Diagnosis

A differential diagnosis was conducted to consider other dermatologic conditions, namely erysipelas, erysipeloid, *granuloma annulare*, lichen sclerosus, morphea, lipodermatosclerosis, systemic sclerosis, and thinning of skin due to aging. However, these dermatologic entities did not imitate or match the rashes presented by the five patients. Sclerotic lesions in patients with ACA are frequently diagnosed as morphea or lichen sclerosus [27]. Many rashes exhibited by Lyme disease patients are incorrectly considered insect or spider bites, or other dermatologic conditions, and dismissed as being insignificant. The preliminary ACA manifestations are often clinically overlooked or misdiagnosed, and attributed to circulatory problems or aging [27]. For example, a blister rash has been frequently mistaken for poison ivy, allergic reactions, and herpes simplex [56]. In addition, Berger et al. demonstrated that punch skin biopsy specimens taken from the peripheral border of EM rashes cultured positive in 18 (86%) of 21 specimens and, notably, two of these EM lesions were less than 5 mm in diameter [57]. Similar to EM rashes, ACA rashes change intensity of color, and disappear as spirochetes disseminate by hematogenous or lymphatic spread from the tick-bite site. Whether a tick bite is recalled or not, an ACA may appear at the tick bite centrum. A bluish-red discoloration, often with swelling and/or atrophy of skin on an apical or lower area of an extremity, together with positive Bbsl serology or Lyme immunoblot testing, is consistent with ACA [27].

The primary site of ACA involvement on the upper extremities is normally the olecranon area (elbow) or the dorsal aspect of the hands (Figure 3) [27]. The presence of the ACA rash on the hands of Case 3 is also exhibited by other chronic Lyme disease patients in different parts of the world [27]. 

Healthcare practitioners have conjectured that the purple discoloration on the back of the knee is due to bending. However, in Case 1, the ACA on the posterior side of the knee does not correspond to bending, bruising, or exercise. Likewise, in Case 4, the purple rash on the foot and the lower leg is an apparent sign of persistent Bbsl infection. The presence of Bbsl in skin biopsies of Case 4, by PCR, confirms borrelial infection in the cutaneous tissue of this ACA. Additionally, in Case 4, the boy not only had ACA rashes on his right leg and foot, he had clubfeet when he was an infant. Consistent with the findings of Case 4, pathologists and clinicians have reported clubfeet in pediatric cases resulting from gestational Lyme disease [58,59]. As apparent in these pediatric cases, a clubfoot is one of the many physical abnormalities associated with transplacental transmission of Bbsl from mother to fetus during gestation. As an infant, this young patient (Case 4) was fussy and cranky and, in retrospect, was likely responding to infected clubfeet. As an adolescent, Case 4 developed swollen feet and ACA rashes. One foot was corrected to some extent with numerous casts; however, the other foot had to be corrected with surgery. As well, he had to wear orthopedic shoes on both feet for five years. At age 14, this youthful patient was diagnosed with Lyme disease by a clinician specializing in the diagnosis and treatment of tick-borne, zoonotic diseases. Eventually, his symptoms resolved, and treatment was successful. The presence of motile spirochetes positive for *B. burgdorferi* sensu stricto by PCR taken from the leg lesion of Case 4 documents conclusively that this rash is an ACA. 

Using PCR, Moter et al. detected Bbsl spirochetes in skin biopsies of Lyme disease patients, and 92 % of the ACA rashes were positive. Veterinary researchers have shown that dogs inoculated with heat-killed Bbsl spirochetes are cleared from the body within 3 wk and, subsequently, they are not detectable by PCR [60]. Despite standard antimicrobial treatment, viable Bbsl spirochetes can sequester and survive in deep-seated tissue. Clinical researchers have detected Lyme disease spirochetes in testicles [61], semen [41], vaginal secretions [41], and vulvovaginal tissue [62]. At birth, Bbsl has been detected in placental tissue [59,63] and cord blood [59]. These collective findings provide supportive evidence for Bbsl transfer during intimate relations, and transplacental transmission of Lyme disease spirochetes during gestation. In addition, Bbsl spirochetes have been detected in breast milk [64,65]. The collective events relating to Case 4 provide substantive evidence of maternal-fetal transmission of Bbsl and supportive evidence of sexual transmission between two intimate partners.

### 4.5. Ticks as a Source of Bbsl Infection 

Tick bites normally signify the location of a rash for early localized and early disseminated Lyme disease. Moreover, in Case 1, both rashes had a central punctum that is indicative of two separate tick bites; a punctum typically disappears in three weeks. Findings by dermatologists reinforce the fact that reddish-blue discolorations can be anywhere on the leg, including the foot and calf [27]. Flexibility and motion of Lyme disease patients are not a factor in the geographic location, size of lesion, and placement of ACA rashes. Age is not a salient factor; however, ACA rashes tend to appear in older individuals. Based on this study, joint swelling is not associated with the color and size of rashes. As with EM rashes, ACA rashes typically change color, and disappear as spirochetes disseminate from the tick bite or dissipate from the centrum of infection. Glib and hasty assessments of EM and ACA variants can lead to invalid clinical diagnoses and ineffective treatment [27].

In this human study, we assessed five Lyme disease patients, and each patient had lesions consistent with ACA. As a commonality, each participant tested serologically positive for Lyme disease, and each developed an ACA juxtaposed by typical clinical symptoms of Lyme disease. In the absence of antimicrobials, our findings reveal that ACA rashes are long-term, and are consistent with the persistence of Bbsl infection. In addition, we show rashes compatible with ACA may occur regardless of geographic residence. Although two patients may have acquired Lyme disease in Europe, the other 3 cases had no relevant travel to known Lyme disease endemic areas, and must have acquired Lyme disease in Canada. Despite the persistence of chronic Lyme disease [66], this young patient (Case 4) responded well to three years of antibiotic treatment, and clinical signs and symptoms completely resolved.

### 4.6. Clinical Implications of Lyme Disease

Whenever there are delays in diagnosis and treatment, persistence of Bbsl ensues [41,67,68,69,70,71,72,73,74,75,76,77,78,79,80,81,82,83,84,85]. Chronic Lyme disease is capable of inflicting lasting damage [3,86]. About 63% of patients infected with Bbsl develop chronic Lyme disease [87]. Bbsl colonize in many immune-privileged sites, including bone [67], brain [88,89,90], eye [78], collagenous tissues (ligaments, tendons) [68,69], heart [90], kidney [90], bladder [80], liver [90], muscle [91], synovial cells [70], central nervous system [92,93,94], glial and neuronal cells [95,96,97], and fibroblasts/scar tissue [98]. Consistent with syphilis [99], Lyme disease may be an insidious neurologic pathogenesis characterized with demyelination and, ultimately, can be a fatal spirochetosis [74,78,88,90]. Late stage chronic Lyme disease has been defined [3,86,90,100,101], and persistent infection with or without administration of antibiotics is clearly a feature with this tick-borne, zoonotic disease [90]. Furthermore, this stealth pathogen is pleomorphic with diverse forms that include spirochetes, spherocytes (round bodies), blebs, granules and, collectively, biofilms exacerbate the sequestration of Bbsl infection.

Chronic Lyme disease commonly has a complex combination of clinical manifestations [3,101]. In one particular study [102], 73% of patients experienced a Jarisch–Herxheimer reaction after treatment. This persistent spirochetosis commonly causes pronounced disability, and potentially gives rise to central nervous system complications, short-term memory loss, and cognitive impairment [88,90,100,102,103]. Often in chronic Lyme disease, Bbsl invades the bladder wall resulting in abnormal sphincter function, including detrusor hyperreflexia (increased urgency to urinate), detrusor areflexia (urinary void difficulty), and dyssynergia (disturbed muscular coordination) [80]. Based on a cascade of unrelenting pain, incapacitating fatigue, unending depression, inexorable anxiety, indefatigable inflammation, loss of hope and purpose, lack of understanding from school, work, family, and healthcare providers, Lyme disease patients may reach a point of merciless, overpowering despair, and commit suicide [27,104,105,106]. Late stage unremitting meningoencephalomyelitides and all-encompassing Bbsl infection may ultimately result in fatal outcomes [74,78,88,90]. Bbsl may potentially be transmitted to a partner via intimate relations [41,62]. Just as congenital syphilis and related deformities are recognized in pregnant mothers and neonates [99], gestational Lyme disease can present with multiple birth abnormalities in the fetus and infant. These can include clubfeet, miscarriage, spontaneous abortion, stillbirths, and perinatal death [58,59,107,108,109]. Developmental delays, retardation, sudden death syndrome, gestational toxemia, gestational heart disease, as well as failure to thrive, may all occur from unrecognized gestational Lyme disease [59]. The ACA rash provides additional information for healthcare practitioners to make a clinical diagnosis.

## 5. Conclusions

This study reports ACA rashes on Lyme disease patients for the first time in Canada. The author sheds new light on the predominant *B. burgdorferi* sensu stricto genospecies in North America acting as a potential contagion in the pathogenesis of ACA. Although ACA has a predilection for sites on the lower extremities, where collagenous tissue is prolific, ACA has no direct correlation with a tick bite, geographic location, age, exercise, Bbsl genospecies, and location on the skin surface. The ACA lesion is one of a broad variation of different rashes associated with Lyme disease, and denotes that Bbsl infection can be persistent in cutaneous and/or connective tissue. As noted in this study, ACA rashes can last for months or years. Recognition of EM and ACA rashes, and their variants, is clinically important as an indicator in the diagnosis and treatment of Lyme disease. The presence of a co-infection of Bbsl and *Bartonella* sp. in Case 5 displays a novel combination of rashes including an ACA, blister exudate, erythematous eruptions, and *Bartonella* striae. An adolescent male (Case 4) displays an ACA rash caused by *B. burgdorferi* sensu stricto and, with physical deformities (clubfeet) at birth, confirms gestational Lyme disease. Because the filial son (Case 4) of *B. burgdorferi* sensu stricto-positive parents was likewise positive for *B. burgdorferi* sensu stricto, this positivity provides formidable evidence of sexual transmission of Lyme disease spirochetes between intimate partners. Since early treatment of Lyme disease can generally mitigate or prevent late stage clinical manifestations, healthcare practitioners must include ACA rashes in their differential diagnosis for patients showing symptoms compatible with Lyme disease.
